# ERP evidence for Slavic and German word stress cue sensitivity in English

**DOI:** 10.3389/fpsyg.2023.1193822

**Published:** 2023-06-23

**Authors:** Marina Ivanova, Christiane R. Neubert, Josef Schmied, Alexandra Bendixen

**Affiliations:** ^1^Faculty of Humanities, English and Digital Linguistics, Institute of English and American Studies, Chemnitz University of Technology, Chemnitz, Germany; ^2^Faculty of Natural Sciences, Cognitive Systems Lab, Institute of Physics, Chemnitz University of Technology, Chemnitz, Germany

**Keywords:** non-native English, word stress perception, cue weighting, Electroencephalography (EEG), Mismatch Negativity (MMN)

## Abstract

Word stress is demanding for non-native learners of English, partly because speakers from different backgrounds weight perceptual cues to stress like pitch, intensity, and duration differently. Slavic learners of English and particularly those with a fixed stress language background like Czech and Polish have been shown to be less sensitive to stress in their native and non-native languages. In contrast, German English learners are rarely discussed in a word stress context. A comparison of these varieties can reveal differences in the foreign language processing of speakers from two language families. We use electroencephalography (EEG) to explore group differences in word stress cue perception between Slavic and German learners of English. Slavic and German advanced English speakers were examined in passive multi-feature oddball experiments, where they were exposed to the word *impact* as an unstressed standard and as deviants stressed on the first or second syllable through higher pitch, intensity, or duration. The results revealed a robust Mismatch Negativity (MMN) component of the event-related potential (ERP) in both language groups in response to all conditions, demonstrating sensitivity to stress changes in a non-native language. While both groups showed higher MMN responses to stress changes to the second than the first syllable, this effect was more pronounced for German than for Slavic participants. Such group differences in non-native English word stress perception from the current and previous studies are argued to speak in favor of customizable language technologies and diversified English curricula compensating for non-native perceptual variation.

## 1. Introduction

Prosody is crucial for language perception, production, and synthesis, as prosodic elements like word stress, intonation and rhythm enable speech segmentation and understanding (e.g., Cutler, 2015). Word stress fulfils important culminative, contrastive, and delimitative functions (Arvaniti, 2020), as it emphasizes syllables in multi-syllable words, distinguishes between word meanings in lexical stress languages, and segments words in fixed-stress languages. English has variable and lexical stress which is cued through increased pitch, intensity, and duration, shallower spectral tilt and often through changes in vowel quality and glottal parameters (Fry, 1958; Sluijter and van Heuven, 1996; Fuchs, 2016). Cues work as indicators to the perception of information and support its categorization (Toscano and McMurray, 2010) – in this case, as stressed or unstressed. Moreover, cues are combined and integrated to create percepts (Martin, 2016) – the evidence for a particular representation is weighted to determine which representation is activated (Martin, 2016). Word stress cues thus combine with intonation, segmental information, and semantic and pragmatic contextual cues to shape the final perception of a word.

There is strong evidence that English speakers from different language backgrounds weight word stress cues differently (Chrabaszcz et al., 2014; Tong et al., 2014; Meng et al., 2020; Zeng et al., 2022; Zhang et al., 2023). For instance, Cantonese English learners rely more on the pitch than the duration of the syllable in comparison to Mandarin English learners (Meng et al., 2020) and native English speakers (Tong et al., 2014). Compared to Australian English speakers, Taiwanese Mandarin speakers of English are also more sensitive to pitch cues (Zeng et al., 2022). In view of these differences, it is of interest to investigate word stress cue perception in other language groups, as we will do here for advanced Slavic and German learners of English.

Slavic English varieties have great research potential, as word stress shifting and particularly mis-stressing (e.g., ˈcomputer*, ˈprofessor*) is a common feature of Czech and Polish English (Volín and Weingartová, 2014; Porzuczek and Rojczyk, 2017). A study on Czech English production reported that syllable stress was misplaced to the first syllable in about 50% of the stress alternation cases while all other alternatives (i.e., misplacing to the second, third or fourth syllable, erroneous addition or omission of stress) occurred with considerably lower probability (Skarnitzl and Rumlová, 2019). Meanwhile, Polish has even been used to test what was termed as the “stress deafness” hypothesis (Dupoux et al., 1997), now referred to as “stress insensitivity” [see Nikolić and Winters (2022)]. This theory expects speakers of languages with fixed word stress (like Polish, Czech, and Macedonian) and speakers of languages with variable stress (like English, German, Bulgarian, and Serbian) to have varying sensitivity to word stress changes, depending on the amount of lexical exceptions to stress regularity in their native language (Peperkamp et al., 2010). A comparison between Polish, French, Finnish, Hungarian, and Spanish stress perception showed that Polish speakers have only intermediate issues with processing word stress due to the amount of exceptions to the default penultimate stress in Polish (Peperkamp et al., 2010). Domahs et al. (2012) used behavioral and electroencephalography (EEG) measures to show that this difficulty is realized depending on whether the stress is shifted to or from a default or exceptional (non-default) position. Polish stress insensitivity also differed depending on the measurement method – the behavioral stress judgment test had a high error rate (i.e., participants did not actively report perceiving the stress to be incorrect) while EEG showed indirect processing of stress violations (Domahs et al., 2012). Still, changes to the non-default stress were more salient both in the EEG and in the behavioral data (Domahs et al., 2012). Hence, Polish speakers have been observed to be only partially stress-insensitive. While both Czech and Polish are fixed-stress languages, issues with word stress extend to other Slavic languages as well – e.g., mis-stressing was found in Serbian speakers of English too (Đurović and Silaški, 2013). All in all, studying word stress perception has potential for generating insights that could inform the development of language acquisition strategies and technologies to support Slavic foreign language learners.

Specifically looking at which cues convey word stress to speakers of Slavic languages, there is limited evidence and only from Russian. In a behavioral stress identification task of the nonword *maba*, Russian speakers were shown to rely on the vowel quality, intensity, and duration of the syllable, but not that much on the pitch (Chrabaszcz et al., 2014). However, these results may not be generalizable to other Slavic languages and cue perception in a foreign language. Russian as an East Slavic language is different from West and South Slavic languages in terms of word stress production and particularly vowel reduction – for instance, in contrast to Czech and Bulgarian English speakers who under-reduce vowels (Stoykova, 2018; Volín and Johaníková, 2018), Russian English speakers have been shown to over-reduce since Russian allows one prominent syllable per word (Banzina et al., 2016).

Due to the word stress differences of East Slavic languages compared to West and South Slavic languages, the current study focuses on speakers from West and South Slavic backgrounds.^1^ These varieties are underresearched, as studies do not focus explicitly on foreign language perception but on native language or pseudoword perception, or they investigate production rather than perception cues. Pitch was found to be a strong cue in Polish and English (Jassem et al., 1968; Dogil and Williams, 1999), whereas duration was more effective with Polish (Jassem et al., 1968). The crucial place of pitch in Polish stress perception has led to its description as a “melodic or tonal” language (Jassem, 1959, p. 269). A later acoustic study found that intensity was the most prominent acoustic correlate of Polish stress followed by pitch and duration (Łukaszewicz and Rozborski, 2008). Yet, the analysis was carried out only on vowels and the results may differ in the context of the syllable. Malisz and Wagner (2012) found pitch, duration and intensity to be equal correlates to stress in Polish conversation and confirmed the widespread view of the Polish stress system as rather “weak” (Dogil and Williams, 1999, p. 284). When pitch was held constant, Polish English learners were able to use vowel duration and vowel quality as stress cues (Rojczyk, 2013). A study on duration in vowel perception showed that Czech speakers rely on vowel duration for discriminating both native and non-native (Estonian) vowels (Chládková et al., 2013). Duration and intensity were generally found to be stable perceptual cues to Czech stress (Janota and Liljencrants, 1969) and pitch has been identified as a strong predictor of stress in a neural network-based model (Duběda and Votrubec, 2005). Yet, in casual speech production, stressed vowels did not exhibit a significant increase in pitch, duration or intensity (Skarnitzl, 2018). Production studies show that Czech English stress mostly differs from British English in terms of intensity, followed by pitch, spectral tilt, and duration (Volín and Weingartová, 2014), i.e., Czech English speakers were able to use duration most natively and pitch and intensity least natively. Weingartová et al. (2014) also concluded that Czech English speakers did not use word stress cues to distinguish stressed and unstressed syllables in English production as systematically as native speakers. Without focusing on stress, Andreeva and Dimitrova (2022) demonstrated that Bulgarian English has a higher pitch level and span than native English, confirming the importance of pitch found for native Bulgarian word stress production (Dimitrova, 1998b). Duration and pitch were also strong cues to Bulgarian English stress perception (Dimitrova, 1998b). Word stress in Serbian is marked by relative duration, yet, since it falls on the syllable with a high tone, it also correlates with pitch (Lehiste and Ivić, 1986). In addition, spectral tilt was shown be an acoustic correlate to stress in Polish, Macedonian, and Bulgarian (Crosswhite, 2003), however, this stress correlate has not been reliably established yet (Gordon and Roettger, 2017) and we will thus only focus on pitch, intensity, and duration. West and South Slavic languages are also specific in terms of rhythmic class, as they are placed on a continuum between stress-timed and syllable-timed languages (Dimitrova, 1998a; Dankovičová and Dellwo, 2007; Marković and Milićev, 2011; Gralińska-Brawata, 2015). The division into stress-and syllable-timed languages is based on a set of parameters in which languages differ and this classification has prompted many discussions (review in Mołczanow and Wiese, 2014), but what unites South and West Slavic speakers is their full pronunciation of vowels in unstressed syllables (e.g., Dimitrova, 1998a; Dankovičová and Dellwo, 2007). Even in Bulgarian, which has vowel reduction and should be able to weaken the prominence of unstressed syllables, changes in vowel quality and duration in unstressed positions take place to a smaller extent compared to stress-timed languages like English (Dimitrova, 1998a) and Bulgarians face issues with English vowel reduction (Stoykova, 2018). When it comes to stress variability, West Slavic languages have fixed stress – Czech on the first syllable and Polish on the penultimate (with groups of exceptions, see Domahs et al., 2012), whereas South Slavic languages are more mixed – Bulgarian has variable stress, Macedonian has fixed antepenultimate stress, whereas Serbian has variable stress and lexical tone. Still, there is not enough evidence to construct hypotheses about the individual languages, therefore, for the purpose of gaining first insights on Slavic non-native English word stress perception, we will group all represented languages into one Slavic group.

The second focus of this study falls on German word stress perception in English. German and English are both West Germanic languages with stress-timed rhythm and variable stress. The research on German English word stress perception is again scarce. Germans are sensitive to suprasegmental differences, as participants in a forced-choice identification task successfully used suprasegmental cues for stressed and unstressed syllables (Yu et al., 2020). Word stress processing research has mostly focused on metrical structure, pointing at large interindividual differences in prosody processing (Heisterueber et al., 2014). In production, duration followed by pitch, intensity, and vowel quality have been identified as the main acoustic correlates of German stress (Jessen et al., 1995).

There are few empirical studies comparing Slavic and German word stress production and perception. In Dogil and Williams (1999), duration was a significant acoustic cue in German even outside intonational focus, unlike pitch in Polish. A comparison of German, Russian, Czech, and Polish word stress in Graupe (2011) showed that Polish speakers tend to use duration as a production cue, yet the tendency for Czech and German is unclear. In perception, speakers of Russian and German were more successful in the behavioral task on word stress placement differentiation than speakers of Czech and Polish, probably due to Russian and German’s variable and distinctive stress (Graupe, 2011). Still, these results are based on an experiment involving pseudowords and it is unclear whether they can be directly applied to the perception of foreign languages like English.

The comparison of Slavic and German English word stress perception is interesting because both groups are learners of English, yet from different language families, where German and English are from the same language family and are expected to have more similar stress perception. However, studies on Slavic and German word stress perception in English as a foreign language are scarce and their evidence is mixed. There are also hardly any direct comparisons between Slavic and German English speakers, thus, it is uncertain whether the previous findings are specific for the used stimuli. Overall, the noticeable gaps in the research on non-native English word stress cue perception hinder the formulation of sound hypotheses. The current study is therefore of exploratory nature and is the first to provide a direct comparison of Slavic and German word stress cue perception in English.

We aim to address the existing gaps in non-native word stress perception by using brain responses as measured by electroencephalography (EEG). The Event-Related Potentials (ERPs) time-locked to stimulus presentation can provide an indirect measure of cue perception independent of attention. To investigate the detection of unexpected auditory information in non-linguistic and linguistic sounds, an ERP component named Mismatch Negativity (MMN) is widely used (Näätänen et al., 2007; Garrido et al., 2009). The MMN is usually elicited in oddball paradigms, where participants are presented with many frequent stimuli (standards) and few infrequent and thereby unexpected stimuli (deviants). MMN elicitation depends on extracting the standard sounds’ regularity (predictability) and noticing deviations from this regularity, i.e., prediction violations (Näätänen et al., 2007; Garrido et al., 2009). MMN is elicited both when participants pay attention to the stimuli and when their attention is diverted from the stimuli, such as by having them watch a silent movie. The latter approach is called passive listening and serves to prevent the elicitation of confounding attention-dependent ERPs like the N2b (Novak et al., 1990). The MMN peaks at about 100–250 ms after the onset of the deviation and can be observed as negativity at frontocentral scalp locations with polarity inversion at the mastoid electrodes if the reference electrode is placed at the nose (Schröger, 1998). The MMN amplitude is interpreted to reflect the amount of predictability violation, which is a function of the degree of unexpectedness (relative to the extracted regularity) and the salience of the sensory mismatch, at least for small (near-threshold) deviations (Horváth et al., 2008). The fact that more salient deviations lead to higher MMN amplitudes can be exploited to measure individual-or group-level salience of certain deviations (Kujala et al., 2007). This approach lends itself to research on language perception by different groups, where for instance familiarity with the speech sounds modulates MMN amplitude (e.g., Jacobsen et al., 2004; Ylinen et al., 2009). In addition, the component’s latency can also be an indicative feature, for instance, in the detection of phonotactic combinations supporting or contradicting listeners’ experience from their native language (Emmendorfer et al., 2020). Thus, the MMN integrates top-down predictions with bottom-up input (Bornkessel-Schlesewsky and Schlesewsky, 2019) and can be used to explore how speakers from a certain language group perceive fine acoustic differences.

Word stress cues are an example of acoustic differences which can be weighted differently. For instance, Zora et al. (2015) examined the perception of English words and pseudowords with word stress cued through pitch, intensity, or a combination of both. Early processing of prosodic information in words was indexed by an intensity-related MMN deflection and a P200 related to fundamental frequency (F0) (Zora et al., 2015) whereas pseudowords elicited only a late MMN. The study concludes that the absence of early potentials for pseudowords and the presence of early and larger ERP responses for words cannot be attributed only to simple acoustic change detection but is probably a result of pre-existing memory traces for prosodic information (Zora et al., 2015). The lexicality effect of MMN has, however, been questioned since (Politzer-Ahles and Im, 2020).

The observation of more than one component at the event of stress changes as in Zora et al. (2015) is not rare. Honbolygó et al. (2004) and Honbolygó and Csépe (2013) examined the perception of words and pseudowords in Hungarian and found that the deviant with an illegal stress pattern elicited two MMNs while the deviant with a legal pattern elicited no MMN. The results were interpreted in the frame of a perceptual process where input is compared both between short-term acoustic traces and a long-term stress template, resulting in the stronger early N2 followed by the MMN component (Honbolygó and Csépe, 2013). A similar MMN-LDN (Late Discriminative Negativity) sequence in response to word stress cues was observed in Honbolygó et al. (2017). Two MMNs were elicited in Chung and Bidelman (2016) who looked at neural tracking of native vs. non-native (Mandarin) speakers of English. Native speakers showed more robust encoding and tracking of the amplitude envelope of speech (Chung and Bidelman, 2016) whereas non-native speakers showed smaller MMN amplitudes when primary stress placement was changed across multiple syllables (Chung and Bidelman, 2016). While intensity was used more by English native speakers, there was no significant difference between the groups in their use of pitch (Chung and Bidelman, 2016).

Word stress cue perception has been extensively examined in studies on East Asian English varieties like the one by Chung and Bidelman (2016). Tong et al. (2014) studied the perception of Cantonese children learning English by exposing them to the word *mother* with original and manipulated stress based on cues extracted from the word *today*. In the 170–270 ms window, they observed a pitch-related positive mismatch response (p-MMR, note that finding positive rather than negative mismatch responses is not uncommon in children) as well as a duration MMN, whereas in the 270–400 ms window, they observed an intensity p-MMR and MMN elicited by the combination of all three cues (Tong et al., 2014). This led to the conclusion that cues can vary with the unfolding process of stress perception (Tong et al., 2014). This study was replicated by Meng et al. (2020) with focus on Cantonese and Mandarin English learners. Using the same stimuli and paradigm, they found that Cantonese speakers rely on pitch information even in the condition when all three cues were varied simultaneously (Meng et al., 2020). Both for the Mandarin and the Cantonese English learners, pitch was perceived as a stronger cue than duration and intensity (Meng et al., 2020), yet the Cantonese weighted pitch stronger than the Mandarin English learners. This result was explained by the higher number of tones in Cantonese, requiring higher sensitivity to pitch (Meng et al., 2020). A follow-up study focusing only on pitch confirmed that Cantonese English speakers use pitch more than Mandarin English speakers for both word stress perception and word recognition in English (Zhang et al., 2023). Zeng et al. (2022) tested two theories on cue weighting during stress perception – segmentation based on the rhythmic properties of the native language or based on the iambic-trochaic or strong-weak grouping of sounds (Zeng et al., 2022). They found a language-specific familiarity effect (Zeng et al., 2022) in the perception of native and non-native English speakers. The MMN and LDN also provided evidence for a trochaic bias in the native English and in the Taiwan English group, which was attributed to the English language skills of the non-native speakers (Zeng et al., 2022).

Overall, previous research confirms that foreign language word stress perception differs based on the language background, and that the MMN component is a suitable tool for tapping into such differences. Here, we extend this research to other language families, specifically to word stress perception in English by Slavic and German participants. The results will be interpreted in a general language cognition framework and the relevance of this research will be illustrated by possible applications in the design of language technologies.

## 2. Materials and methods

### 2.1. Stimuli and paradigm

We employed an oddball paradigm with the English word *impact*, which exists in two forms – as a noun with stress on the first syllable (*ˈimpact*) and as a verb with stress on the second syllable (*imˈpact*).^2^ The word pair *ˈimpact*–*imˈpact* was chosen because it is contrasted only by stress, similar to *upˈset*–*ˈupset* used in Zora et al. (2015). Word stress shifts in English often combine suprasegmental changes with segmental changes in vowel quality (e.g., Burzio, 2007), which would act as a confounding variable. The stress shift in *impact* also does not cause a change in meaning, which aims to avoid lexical violations. The word pair, however, differs in word class and word frequency – the noun *ˈimpact* occurs 105,330 times in the Corpus of Contemporary American English (Davies, 2008) whereas the verb *im*ˈ*pact* occurs 9,804 times. This follows the typical stress distribution in English, as about 80% of English disyllabic words have first syllable stress (Howard and Smith, 2002). The choice of *impact* thus aims to reduce potentially contradictory effects of global word frequency and more local word class frequency, as it would have been the case with a word pair where the more frequent word is stressed on the second syllable, for instance *inˈcrease-ˈincrease*.

**Figure 1:**
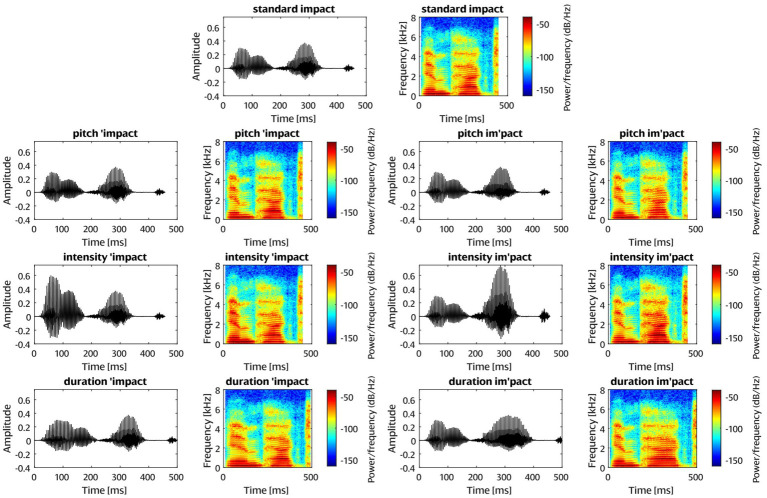
Sound waveforms and spectrograms of the standard and deviant stimuli employed in the current study.

The neutral version of the word *impact* was synthesized using Balabolka (Morozov, 2022). The synthesis was carried out with flat intonation to allow for comparable pitch manipulations of the two syllables in the two stress positions. The word was split into its syllables and their pitch, intensity, and duration were manipulated in Praat (Boersma and Weenink, 2022). In determining the amount of manipulation on each cue, we aimed for equal salience of the three cue types. Studies on cue perception face the challenge of determining whether the results are based on language-specific cue perception or on the salience of the particular cue (Chrabaszcz et al., 2014; Zeng et al., 2022). To arrive at similar cue salience, we compared the manipulations in previous similar studies (Table 1). On this basis, syllable pitch was increased by 10%, syllable intensity was increased by 6 dB and vowel duration was increased by 1.7, leading to a vowel duration increase of about 68% and syllable increase of 20% in the first and 25% in the second syllable. Only the vowel duration was increased in the duration condition to avoid artificially prolonged consonants. The manipulations only ever concerned one cue; we did not employ double or triple manipulations. The manipulated syllables were concatenated back into words in MATLAB (MathWorks, 2022). This resulted in altogether seven stimuli: neutral *impact*, three versions of *ˈimpact* (with pitch, intensity, or duration increase on the first syllable), and three versions of *imˈpact* (with pitch, intensity, or duration increase on the second syllable) (Figure 1). When the first syllable was manipulated, the second syllable was identical to that in the neutral word, and vice versa. The end of each word stimulus was adjusted with an offset ramp to avoid distortions.

**Table 1:** Comparison of duration, intensity, and pitch values in original and manipulated stimuli in this study and previous research on word stress cue perception.

Study	Stimulus	Deviant syllable	Standard			Deviant			Increase		
			*P*.	*I*.	*D*.	*P*.	*I*.	*D*.	*P*.	*I*.	*D*.
Current study	/ˈɪmpӕkt/	first	210.53	73.00	176	231.88	79.00	220	10%	6.0	25%
Current study	/ɪmˈpӕkt/	second	222.22	71.00	282	246.15	77.00	339	10%	6.0	20%
Meng et al. (2020)	/ˈmʌðɚ/	first	216.70	76.78	148	165.61	69.35	98	−23%	−7.4	−34%
Meng et al., 2020	/mʌˈðɚ/	second	180.20	74.48	152	202.08	78.38	228	12%	3.9	50%
Zeng et al. (2022)	/dede/	each	190.00	65.00	180	209.00	71.00	239	10%	6.0	33%
Honbolygó et al. (2017)	/ˈnɒnɒ/	first	141.30	71.00	98	152.30	74.30	133	7%	3.3	35%

D., duration (ms); I., intensity (dB SPL); P., pitch (Hz). The presented pitch and intensity values for this study are the median of the syllable values. For better comparability with the other studies, the syllable duration is presented although the vowel duration was manipulated. The /ɪ/ vowel duration was increased from 63 ms to 106 ms (68%), the /ӕ/ vowel duration was increased from 80 ms to 134 ms (68%).

**Figure 2:**
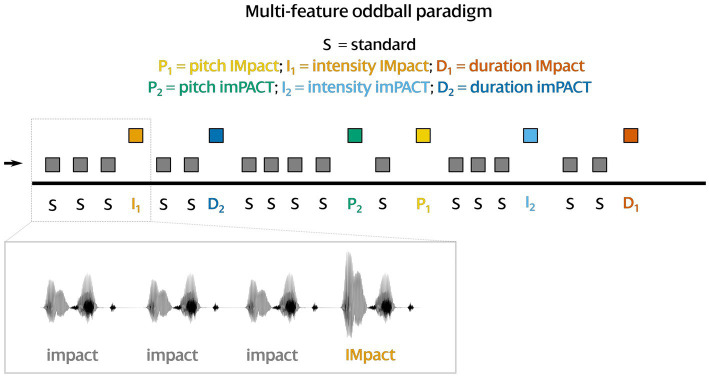
Visualization of the multi-feature oddball paradigm applied in this study. At least one unstressed standard is enforced before each of the deviants. Inter-stimulus interval is jittered within a range of 330 and 630 ms.

The standard (neutral *impact*) and the six different deviants were incorporated in a passive multi-feature oddball paradigm with 3,120 standards (74%) and 1,080 deviants (13% *ˈimpact* and 13% *imˈpact*), i.e., 180 deviants per each of the six types (Figure 2). One standard was enforced after each deviant and one standard at block onset; apart from these restrictions, deviants were interspersed randomly into the stimulus sequence. The overall sequence was organized into 10 blocks, each of which contained 312 standards (neutral, unstressed *impact*) and 108 deviants. The deviant types (pitch *ˈimpact*, intensity *ˈimpact*, duration *ˈimpact*, pitch *imˈpact*, duration *imˈpact*, intensity *imˈpact*) were distributed equally across the blocks, i.e., there were 18 deviants per type in each block. The stimuli were presented with an interstimulus interval (offset-to-onset) of 480 ms on average, randomly drawn from a range between 330 and 630 ms. The duration of each block amounted to 7 min.

### 2.2. Participants

The necessary sample size was calculated through an *a priori* power analysis with GPower (Faul et al., 2007) based on the effect size *d* = 1.033 determined from Meng et al. (2020), with an alpha level of 0.05 and a power of 0.80, which resulted in a total sample size of 32 with two independent groups of 16 participants. We recorded data from 16 German and 16 Slavic (3 Czech, 3 Polish, 2 Serbian, 4 Macedonian, 4 Bulgarian) fluent English speakers (25 females, 7 males) aged between 19 and 38 (*M* = 26, *SD* = 4.5). The participants reported normal hearing and fluent English skills. English level was estimated through years of formal English education (*M* = 12 years, *SD* = 5.06), with no significant difference between the groups [German group *M* = 10.94, *SD* = 3.60; Slavic group *M* = 13.47, *SD* = 6.12; *t* (29)^3^ = −1.414, *p = 0*.1681, *d* = −0.495]. In addition, most of the participants reported English level at B2–C1 following the Common European Framework of Reference for Languages (CEFR) – two participants reported B1, 14 reported B2, 12 reported C1 and four reported C2 level. The participants also reported time spent abroad in English-, German-and Slavic-speaking countries. All experimental procedures were in accordance with the Declaration of Helsinki and were approved by the Ethics Committee of Chemnitz University of Technology (case no. 101560561). The participants provided written informed consent for study participation and data processing, and they received monetary compensation or course credit for their participation.

### 2.3. Experimental procedure

#### 2.3.1. Procedure and EEG recording

Prior to the measurement, the participants filled out a short demographic questionnaire. EEG was recorded with a stationary 64-channel EEG system (Brain Products GmbH) with active AgCl electrodes. The electrodes were placed on an elastic cap with conductive gel and positioned according to the 10–20 system with Fpz as ground, including electrodes at the mastoids, four EOG electrodes for extracting eye movement artifacts and an electrode on the nose for reference. Impedances were kept below 20 kΩ. Participants sat comfortably inside an electrically shielded and acoustically attenuated chamber (IAC Acoustics, Niederkrüchten, Germany) and watched a silent movie while hearing the stimuli presented binaurally through headphones. The EEG signal was recorded with the BrainVision Recorder software (Brain Products GmbH) and sampled at 500 Hz with a 249 Hz online lowpass filter to avoid aliasing. The experiment lasted 70 min and together with the preparation and the breaks between each of the ten blocks, the data acquisition of one participant lasted about 150 min. After the experiment, the participants were asked to report (1) what stimuli they had heard, (2) whether they had noticed anything about the stimuli and the way they were pronounced, (3) whether the three variants *impact*, *ˈimpact* and *imˈpact* exist in English, and (4) whether they know the difference in meaning between *ˈimpact* and *imˈpact*.

#### 2.3.2. Pre-processing

The EEG data were analyzed with EEGLAB 2022.1 (Delorme and Makeig, 2004) in MATLAB R2022b (MathWorks, 2022). The data were first prepared for Independent Component Analysis (ICA) – they were filtered with a 1 Hz highpass filter (filter order: 9056, Kaiser β: 5.6533, passband ripple: 0.001 (−60 dB), transition bandwidth: 0.2 Hz), separated in artificial epochs of 1 s, and non-stereotypical artifacts were excluded. Non-stereotypical artifacts were identified by EEGLAB’s functions *rejkurt* and *jointprob* with thresholds of 3 STD. These settings were used only for the ICA. The decomposed components were independently evaluated by two of the authors (MI, CN) with assistance from an automatic categorization with IClabel (Pion-Tonachini et al., 2019). Components containing stereotypical artifacts like eye, muscle, and heartbeat artifacts and channel noise were removed. This led to the exclusion of 6.6 components per participant on average (*SD* = 1.4). The EEG data were then filtered with a Kaiser-windowed bandpass FIR filter (0.1–45 Hz, filter order: 9056, Kaiser β: 5.6533, passband ripple: 0.001 (−60 dB), transition bandwidth: 0.2 Hz) and segmented into epochs from −100 to 600 ms relative to stimulus onset. The epochs were baseline-corrected using the interval from −100 to 0 ms relative to stimulus onset. Epochs with amplitude change above 100 μV on any electrode were rejected, which left 92% remaining epochs on average (*SD* = 6.05%, *Min* = 74%). The data were then averaged into single-subject and grand averages per stimulus type. The difference between the standard and the deviant waveforms was calculated by subtracting for each participant the ERP elicited by the standard from the deviant ERP, separately for each of the six deviant types. Note that the same standard ERP was used for each subtraction; thus, the statistical information when comparing the difference waves is the same as when comparing the deviant ERPs. Yet applying the subtraction supports the identification of ERP components.

#### 2.3.3. ERP data analysis

**Figure 3:**
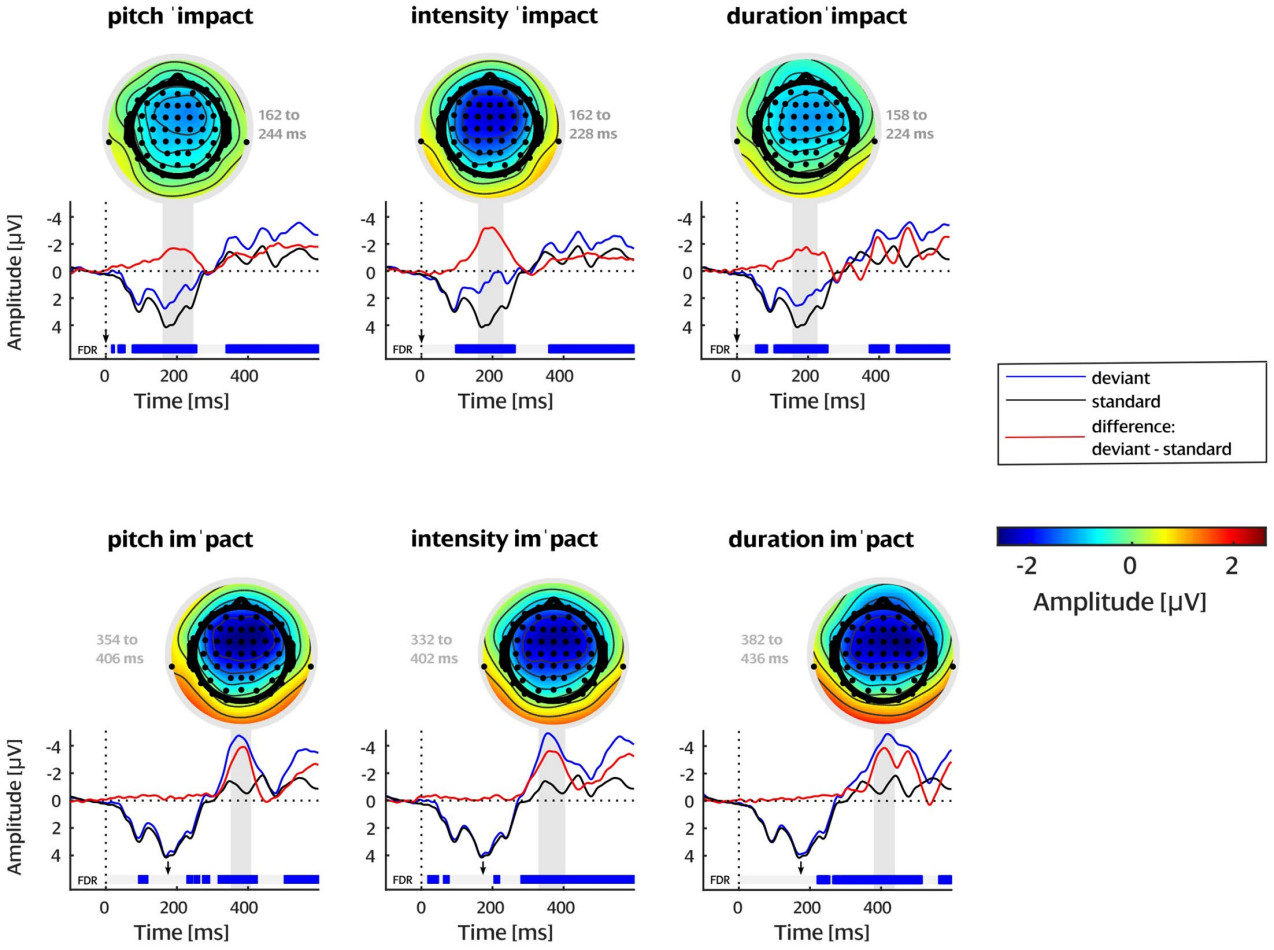
Grand-average topographies with nose reference and ERP waveforms at FCz referenced against average mastoids for each word stress condition. The results from running *t*-tests with FDR correction are presented below the ERP waves with blue regions indicating significant negativity. MMN windows are highlighted with grey rectangles. Arrows point at the beginning of the syllable containing the deviation.

As recommended in MMN research (e.g., Schröger, 1998), all analyses were conducted on the data from FCz re-referenced against the average of the left and right mastoids to capture the full MMN component amplitude including its polarity reversal. First, to verify that MMN was elicited, the difference wave for each deviant type was tested for statistically significant deviations from zero by means of a sample-wise running *t*-test throughout the whole epoch window (i.e., from 0 to 600 ms), correcting for multiple comparisons (i.e., type I error inflation arising from the many consecutive tests) via the false discovery rate (FDR, Benjamini and Hochberg, 1995; Matlab function by Gerber, 2023). This avoids the circularity of component verification through visible peaks (see, e.g., Luck and Gaspelin, 2017). After confirming a significant frontocentral negativity with this procedure, the interval for the MMN analysis was set separately for each deviant type to accommodate for latency differences. In the difference wave for each deviant type, the MMN peak amplitude was determined, and the start and end of the MMN analysis window was set as the timepoint at which 70% of the peak amplitude was passed. This resulted in slightly different window lengths, accounting for the different shapes of the MMN components per deviant type. The final MMN windows for each deviant type are highlighted in the grand average plots and labeled above the topographies (plotted with the original nose reference) in Figure 3. It should be noted that while peak-picking procedures are being criticized for their circularity (“double dipping”; Kriegeskorte et al., 2009; Luck and Gaspelin, 2017), here we chose the latency range for MMN analysis *after* having determined that a significant negativity was elicited for every data sample in that range, and we used it for MMN amplitude quantification and group comparisons. Moreover, it is important to point out that we determined the MMN analysis windows for the whole participant sample, prior to looking at any data split by language group.

After having thus set the MMN analysis windows per deviant type, we quantified MMN mean amplitude (averaging across all datapoints in the analysis window) in the single-subject difference waves. Possible group differences in the processing of the cues were examined in a three-factorial mixed analysis of variance (ANOVA) of mean MMN amplitude with the factors Language group (between-subject, 2 levels: Slavic vs. German), Stress cue (within-subject, 3 levels: pitch/ intensity/ duration), and Stress position (within-subject, 2 levels: first vs. second syllable). The statistical analyses were conducted in R via RStudio (RStudio Team, 2021; R Core Team, 2022) with the core *stats* package, the *rstatix* package (Kassambara, 2022), and the *tidyverse* (Wickham et al., 2019).

## 3. Results

The FDR-corrected running *t*-tests revealed significant MMN components for all six deviant types (Figure 3). The tests also indicated later significant components beyond the MMN latency range, which are quite common with linguistic stimuli, as will be discussed below. Here we focus on the MMN component. All MMN windows determined by 70% peak amplitude (see above) are well in line with the typical MMN latency range (within 160 to 240 ms following stimulus onset for first-syllable deviations, within 160 to 230 ms following deviation onset for second-syllable deviations, taking into account that the deviation starts at 175 ms after stimulus onset for pitch and intensity, and at 225 ms after stimulus onset for duration). Note that duration deviants started to differ from the standard well before the end of the vowel, as is typical of natural language: the vowel contains additional cues such as spectral and pitch cues as to whether it will be short or long (e.g., Lehnert-LeHouillier, 2010). All topographies are in line with the expected MMN voltage distribution, showing a frontocentral negativity with inversed polarity at the mastoids, which is consistent with a generator of this component in auditory cortex (Näätänen et al., 2007).

**Figure 4:**
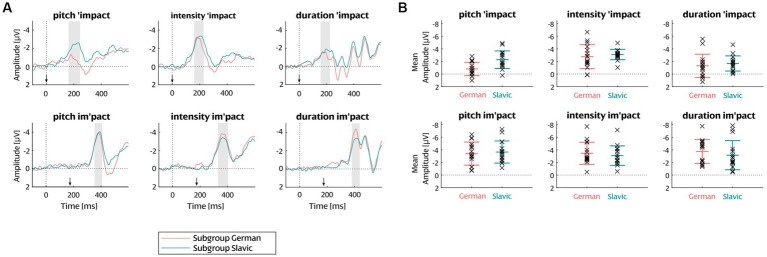
**(A)** Grand-average ERP difference waves at FCz referenced against average mastoids for each word stress condition. MMN windows are highlighted with grey rectangles. Arrows point at the beginning of the syllable containing the deviation. **(B)** Mean and single-subject MMN amplitudes per language group and word stress condition. Each cross indicates one participant; group mean is plotted with error bars reflecting standard deviation across participants.

After confirming MMN for the whole participant sample, MMN amplitudes were read out separately for the German and Slavic subgroups and were compared between them. One-sample t-tests against zero separately for each group and deviant type in the respective MMN analysis windows confirmed that significant MMN was elicited by each cue in each group (all *t* values < −2.87, all *p* values < 0.02). Figure 4 visualizes the difference waves (A) and mean MMN amplitudes (B) of the German and Slavic subgroup per condition.

**Figure 5:**
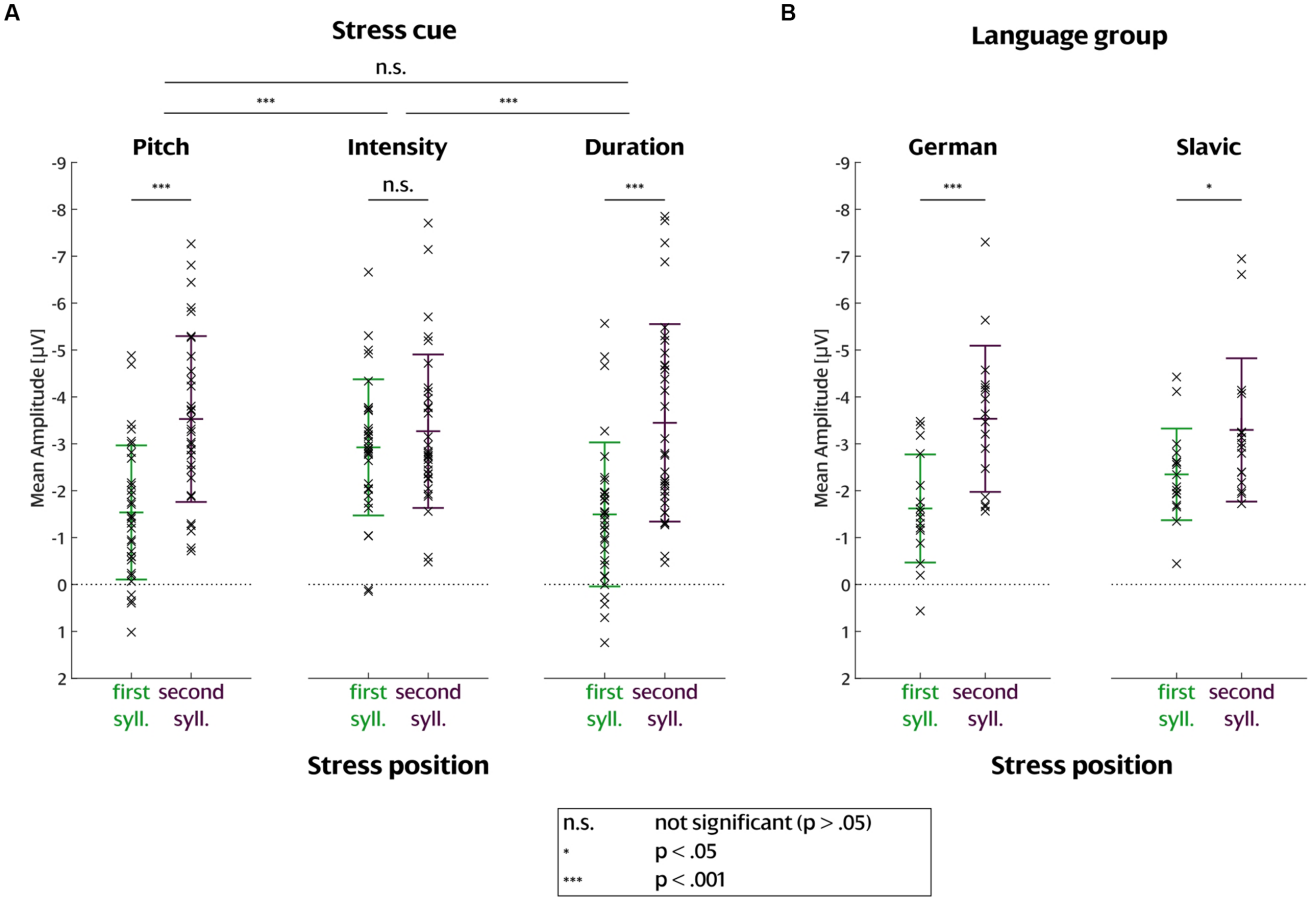
Comparison between mean MMN amplitudes for first and second syllable position **(A)** per stress cue and **(B)** per language group. Each cross indicates one participant; group mean is plotted with error bars reflecting standard deviation across participants. The statistical significance of the difference between the groups is indicated above the respective groups: n.s. *p* > 0.05, * *p* < 0.05, *** *p* < 0.001.

Results of the three-factorial 2 × 3 × 2 mixed-model ANOVA of MMN amplitude are displayed in Table 2. There were significant effects of Stress cue [*F* (2, 60) = 4.594, *p* = 0.014, $\eta_{G}^{2}$ = 0.03] and Stress position [*F* (1, 30) = 68.983, *p* < 0.001, $\eta_{G}^{2}$ = 0.165] as well as an interaction between Stress cue and Stress position [*F* (2, 60) = 10.768, *p* < 0.001, $\eta_{G}^{2}$ = 0.054]. These effects were caused by generally higher MMN amplitudes for deviations on the second than on the first syllable (main effect of Stress position, see Table 3) and by this increase being larger for pitch and duration than for intensity deviants (interaction of Stress cue and Stress position, see Figure 5A). There is a significant difference in the stress position amplitude between pitch and intensity *t* (31) = 4.4167, *p* < 0.001 and intensity and duration *t* (31) = −3.886, *p* < 0.001, however, there is no difference between pitch and duration *t* (31) = 0.095342, *p* = 0.92. This intensity difference is the result of higher amplitudes in the first syllable intensity condition in comparison to the pitch and duration first syllable conditions (Figure 5A). Importantly, the interaction of Language group and Stress position was also significant [*F* (1, 30) = 7.848, *p* = 0.009, $\eta_{G}^{2}$ = 0.022], indicating that the MMN amplitude increase with position was more pronounced in the German subgroup than in the Slavic subgroup (Figure 5B). Separate follow-up t-tests revealed that the MMN amplitude increase with position was nevertheless significant in both language groups separately [German group *t* (30) = 3.9457, *p* < 0.001; Slavic group *t* (30) = 2.0904, *p* = 0.04516] (Figure 5B). No further effects of or interactions with Language group were observed. The absence of significant interactions involving the factors Language group and Stress cue indicates that there is no statistical evidence for a differential processing of the cues by the two language groups.

**Table 2:** Results of a repeated-measures Analysis of Variance (rmANOVA) of grand average difference amplitudes in MMN windows with the factors language group, stress cue, and stress position.

Effect	DF effect	DF error	*F*	*p*	Significance	$\eta_{G}^{2}$
Language group	1	30	0.314	0.579		0.006
Stress cue	2	60	4.594	0.014	*	0.03
Stress position	1	30	68.983	2.86*10^−09^	***	0.165
Language group: stress cue	2	60	2.804	0.069		0.018
Language group: stress position	1	30	7.848	0.009	**	0.022
Stress cue: stress position	2	60	10.768	0.000101	***	0.054
Language group: stress cue: stress position	2	60	0.211	0.81		0.001

Asterisks in the Significance column denote statistical significance: *p < 0.05; **p < 0.01; ***p < 0.001.

**Table 3:** ERP grand average amplitude means and standard deviations at FCz rereferenced against average mastoids per language group and stress position.

Language group	First syllable *M* (*SD*) in μV	Second syllable *M* (*SD*) in μV	Difference *M* (*SD*) in μV
German	−1.62 (1.81)	−3.53 (1.80)	1.91 (1.00)
Slavic	−2.35 (1.28)	−3.30 (1.88)	0.95 (0.94)
Overall	−1.98 (1.60)	−3.41 (1.83)	1.43 (1.07)

In order to consider possible explanations for the results in addition to the native language group influence reported above, we conducted three-factorial mixed-model ANOVAs with each potentially influential demographic factor as a between-subject variable or covariate and Stress cue (3 levels) and Stress position (2 levels) as within-subject variables, reflecting the stimulus design. Results from the 4 × 3 × 2 ANOVA on MMN amplitude with English CEFR level as the between-subject factor (4 levels: B1, B2, C1, or C2) showed that MMN amplitude does not differ based on the participants’ English level [*F* (3, 28) = 1.313, *p* = 0.29, $\eta_{G}^{2}$ = 0.069]. English level also did not interact with Stress cue or Stress position (all *p* values > 0.28). Similarly, an ANOVA with years studying English as a covariate indicated that participants’ average MMN amplitude does not differ based on the number of years they have spent studying English [*F* (1, 29) = 0.003, *p* = 0.955, $\eta_{G}^{2}$ = 6.11*10^−05^] and that this covariate does not interact with Stress cue or Stress position (all *p* values > 0.34). A 4 × 3 × 2 ANOVA on MMN amplitude with number of manipulations the participants were able to report after the experiment (4-level factor: stress, pitch, duration, intensity) showed no effect of the reported manipulations [*F* (3, 28) = 0.558, *p* = 0.647, $\eta_{G}^{2}$ = 0.031]. Again, the number of reported manipulations did not interact with Stress cue or Stress position (all *p* values > 0.67). Finally, a 4 × 3 × 2 ANOVA on MMN with the number of meanings of *impact* reported by the participants (4-level factor: none, only noun, only verb, or both) indicated that the reported knowledge of the stimulus meanings did not affect MMN amplitude [*F* (3, 28) = 0.095, *p* = 0.962, $\eta_{G}^{2}$ = 0.006] and did not interact with Stress cue or Stress position (all *p* values > 0.05). Based on these results, we infer that language group and stress position are the core factors influencing word stress perception in this study.

## 4. Discussion

### 4.1. Slavic and German word stress sensitivity in English

Taking MMN as a proxy of perceptual salience, this study demonstrated that both Slavic and German advanced speakers of English are sensitive to word stress changes to the first and the second syllable cued through suprasegmental manipulations of pitch, duration, and intensity. This is an important finding on Slavic word stress perception in light of behavioral and EEG evidence on Polish partial stress insensitivity toward stress violations in native trisyllabic words (Domahs et al., 2012). Since speakers of different Slavic languages were grouped in our sample, a replication experiment comparing speakers of fixed-and variable-stress languages would be useful to confirm the impact of native stress regularity on cue perception in a foreign language. German English speakers were able to perceive all word stress changes and cues, as expected from their overall sensitivity to suprasegmental changes in English (Yu et al., 2020).

Stress position was a significant factor of stress cue perception both as a main effect across all conditions and in interaction with language group. We focus on the following explanations for the more negative MMN amplitudes for changes in second compared to first syllable: (1) Predictive-coding-based explanation: the effect comes from a stronger acoustic prediction for the second than the first syllable, because the fact that “im” is identical to the standard stimulus generates a strong expectation that a standard stimulus will come. Indeed, there is evidence that the auditory system compares incoming stimuli on a point-by-point basis with the expected template (Grimm and Schröger, 2007), and thus the expectation would be strengthened by the first part of the stimulus for second-syllable deviants. (2) Language-based explanation: the effect comes from (2a) knowledge about typical stress distribution in English, or from (2b) biases about typical stress distribution stemming from the own native language. While more than one explanation may contribute to the overall effect, a purely acoustic predictive-coding mechanism (1) cannot explain the full pattern because it would hold independent of language group (as there is no reason to assume differences in predictive coding between the groups), whereas we find a stronger position effect for German than for Slavic learners of English. The effect being modulated by knowledge of English word stress distribution in an overall global context as well as in more local contexts like word class is possible (2a), but it is unclear why we would expect German learners of English to have a more accurate representation than the Slavic group: the groups neither differed in years of study, nor did we find any effect of proficiency level. Therefore, we consider it most likely that biases about typical stress distribution stemming from the own language background (2b) contribute to the effect of stress position.

German is a stress-timed language and as such, stressed syllables are pronounced with higher prominence relative to the reduced unstressed syllables. Polish, Czech, Bulgarian, Serbian, and Macedonian have all been placed on a continuum between stress-timed and syllable-timed languages (Dimitrova, 1998a; Dankovičová and Dellwo, 2007; Marković and Milićev, 2011; Gralińska-Brawata, 2015) because compared to the stress-timed languages, they pronounce stressed and unstressed syllables more similarly in terms of vowel length and quality (Weingartová et al., 2014; Stoykova, 2018). In turn, it seems reasonable that the Slavic group responded strongly (i.e., showed a relatively high MMN amplitude) to stress increases both when they occurred on the first and on the second syllable, because they are generally less accustomed to stress asymmetries based on their syllable-timed native language. In contrast, the German group is well accustomed to stress asymmetries from their stress-timed native language, and thus their MMN amplitude was more strongly driven by other factors distinguishing between first-and second-syllable stress. This would explain the stronger position effect for German than for Slavic learners of English.

It is also possible that the larger German sensitivity to second-syllable stress (Figure 5B) as well as the overall similar response to intensity deviations regardless of the stress position (Figure 5A) is influenced by an Iambic/Trochaic Law (ITL) effect. This effect was also studied for word stress cue perception in Zeng et al. (2022) and found in both native and non-native (Mandarin) English learners. The ITL describes the grouping of rhythmic sub-patterns during continuous speech (Crowhurst, 2020). It assumes that syllables are rhythmically grouped into trochaic (strong-weak) or iambic (weak-strong) pairs based on their acoustic realization. The law posits that trochaic two-syllable groupings have prominent onsets elicited through higher intensity and pitch whereas iambic two-syllable groupings have prominent endings elicited through higher duration (Hayes, 1995, p. 81; Crowhurst, 2020). German has been shown to have a trochaic organization and to consistently represent the ITL (Bhatara et al., 2013). Trochaic organization is also common for the Slavic languages (Bethin, 1998). Despite the similar trochaic structure of the examined languages, the German sensitivity to iambic shifts may have been strengthened by the language’s stress-timed rhythm, which places even more emphasis on the role of the trochee in word stress. The ITL may also explain the smaller difference between first-and second-syllable stress for intensity than for duration cues, since realizing second-syllable stress (iamb) through higher intensity is a weaker cue to iambic prominence than duration (review in Crowhurst, 2020).

Notwithstanding the difference in effect size between the groups, the position effect was still present for both groups. That is, second-syllable stress deviants elicited a stronger MMN in all conditions. Since both positional deviants *ˈimpact* and *imˈpact* are legitimate words, the current study incorporates no true default and no stress rule violation. Still, based on frequency of occurrence, *ˈimpact* is closer to a default than *imˈpact* (see stimuli section 2.1), and both groups may have been sensitive to this asymmetry. Beyond the specific occurrence difference between *ˈimpact* and *imˈpact*, first-syllable stress is generally more typical than second-syllable stress in English disyllabic words (Howard and Smith, 2002). Yet the same is true for German (Féry, 1998) and for the sampled Slavic languages: Czech, Polish, and Macedonian have fixed first, penultimate and antepenultimate stress, so all disyllabic words should be stressed on the first syllable. In Serbian, the last syllable does not receive high tone and accordingly stress (Lehiste and Ivić, 1986), so disyllabic words would be stressed on the first syllable. In Bulgarian, the penultimate has been indicated as the dominant position (Patseva, 2018), which corresponds to first-syllable stress in a disyllabic word. Since first-syllable stress predominates in all involved languages, it is difficult to separate the influence of (implicit) knowledge about stress distribution in the non-native language (explanation 2a) from internalized stress distribution of the native language (explanation 2b). Still, on the whole, when perceiving word stress shifts, Slavic speakers seem to rely on the frequency of word stress distributions in their native language less than German speakers. This outcome may be due to the characteristics separating the Slavic languages from stress-timed rhythm, such as the relatively similar realization of stressed and unstressed syllables. Since the sensitivity toward weak forms is the main difference between the rhythmic organization of the German, English and Slavic languages, future studies can incorporate EEG in experimental paradigms involving vowel reduction like Yu et al. (2020) to investigate how Slavic vs. German speakers use suprasegmental information in their perception of stress shifts.

One aspect that should be considered in the interpretation of language group differences is the existence of similar words in the examined learner languages, which would be the most straightforward support for explanation 2b above (bias from own language). Since MMN has been shown to be stronger in denotationally meaningful words (Jacobsen et al., 2021), one factor influencing the different response to first vs. second syllable stress may be the lexicality of the syllables, i.e., whether the separate syllables are valid words in the participants’ native languages. For instance, /im/ is a valid contraction in German (*in dem* = *in the_m/n*). In the represented Slavic languages, /im/ only features in Bulgarian and Macedonian as the dative and possessive form of *them*. The English pronunciation of /pækt/ does not feature in the tested languages because /æ/ is not a phoneme in German or in the Slavic languages, but it features with /a/. Considering the word *impact* as a whole, “Der Impact” [ˈɪmpɛkt] exists in German as a loanword from English with a first-syllable stress following the English lexical class stress regularity (see (ii) above). The word is, however, relatively rare (DWDS, 2022a) and half of the German participants did not know the meaning of *impact* in English, making it highly unlikely that their responses were affected by the German loanword. There is also a similar Geology term in German indicating a meteorite impact point, “der Impakt” [ɪmˈpakt], yet this word is very rare (DWDS, 2022b) and equally unlikely to influence the German group’s response. In Bulgarian and Czech, the loanword “impakt” has entered the lexicon with the term “impakt faktor” standing for journal impact factor, yet this use is new, domain-specific, and unlikely to have influenced our participants directly. Altogether, existing similar words in the native language are an implausible explanation for language group differences in the perception of stress position.

Overall, it seems that the rhythmic structure of German and the Slavic languages has influenced the different weighting of first-and second-syllable stress in English and future studies incorporating vowel quality changes can further explore these differences in the language groups. It would also be interesting to juxtapose the general word stress distribution in English (predominantly first stress) and word-class stress distribution (noun = first, verb = second) by examining the perception of words that occur more frequently with second-syllable stress than first-syllable stress, for example *ˈincrease* – *inˈcrease* and *ˈdecrease* – *deˈcrease* (see Davies (2008)). In that way, the less frequent stress with regard to the global word stress distribution in English (second syllable) would coincide with the more frequent stress based on the word class distinction (verb = second; local frequency). Especially if speakers of languages with final syllable stress are tested, the comparison would also allow the knowledge of English word stress regularities (explanation 2a) to be pitted against influence from own language (explanation 2b). The response of speakers accustomed to final stress (2b) toward an English locally frequent (*inˈcrease* as a verb) but globally infrequent (second stress) form (2a) has the potential to indicate which explanation plays the more dominant role in word stress perception.

To our surprise, we did not find striking differences in how the language groups respond to the stress cues (pitch, intensity, and duration). This is unlikely to result from insufficient statistical power, as we did observe group differences regarding the effect of stress position. Our sample size was determined based on the effect size of similar previous studies revealing significant differences in cue processing (Meng et al., 2020, for comparison of Cantonese and Mandarin learners of English), thus – with all due caution – our findings suggest that cue processing differences between Slavic and German stress perception of English words are of lower effect size, if existent. While the previous literature suggested differences between Slavic and German stress *production* in the *native* language (Dogil and Williams, 1999; Graupe, 2011), it is unclear whether these findings apply to *perception* and to a *non-native* language. Even in the native language, perception and production cues do not necessarily overlap – for instance, in English and Dutch, pitch is a stronger word stress cue in perception than in production (van Heuven, 2014). Moreover, Slavic and German perception has not been directly compared, and drawing inferences based on previous individual studies investigating only Slavic or only German groups is not conclusive with respect to cue weighting, both in native and in non-native contexts. This exploratory study was thus the first to indicate that Slavic and German speakers do not use stress cues very differently in non-native English stress perception. However, one caveat of the current study is the advanced English level of the sample – although the participants indicated different CEFR levels (B1-C2, but mostly B2-C1), their ERPs did not differ based on English level, therefore, differences at that proficiency level probably do not play a large role in word stress cue perception. Thus, the results may be influenced by a selection bias – those struggling with English due to the lack of similarity with their native language might be less likely to reach this advanced stage; in turn those who reach the advanced stage, might have overcome the dissimilarity between the stress systems. A similar study with beginners would provide interesting insights on the role of language level on word stress perception.

The observed similarities in cue perception by Slavic and German English speakers may also be motivated by our Slavic sample. Since the study was carried out in Germany, most of the Slavic participants had been exposed to German – 11/16 Slavic participants indicated that they have had a long stay in Germany of more than a year and 15/16 reported speaking German to some extent. Still, there was no significant difference in the mean MMN amplitudes between Slavs who have stayed in Germany and those who have not [*t* (14) = 0.69073, *p* = 0.501]. Another limitation of the sample was that five languages from two Slavic subfamilies were grouped under one Slavic group, generalizing language (e.g., Czech vs. Bulgarian) and dialect differences (e.g., Bohemian vs. Moravian Czech). This decision raises the question of how detailed the grouping of participants in psycholinguistic experiments can get in perception vs. production of native and non-native varieties. This question requires a broader systematic review, which is outside the scope of this study. The current study is the first using EEG to investigate English word stress cue perception of European varieties and starting with more general participant groups allows us to gain first insights on the psycholinguistic processes involved. Having shown that English learners from German and Slavic background indeed respond to different types of stress changes during passive listening, future studies can zoom in on potential differences between individual languages and local varieties.

Looking at the perception of stress cues independent of language group, we observed a main effect of stress cue and an interaction of stress cue and stress position. The most salient stress cue was intensity. Intensity was also the strongest cue for the English group in Zeng et al. (2022), which they found to be a surprising result and explained with an acoustic effect. While an acoustic effect is also possible for our study, the main intensity effect may be seen as qualified by the interaction between intensity and stress position (Figure 5A), as the more negative MMN values for first-syllable intensity make it significantly stronger than pitch and duration. In the current study, responses to first and second syllable stress were found to differ when stress was cued through pitch and duration but not through intensity. Similarly, an intensity MMN was also found in Zora et al. (2015)’s change in stress position from first to second syllable in *ˈupset*-*upˈset*, though based on native English speakers and a full stress shift. One possible explanation for the stronger response to first-syllable stressed intensity deviants is the different place of the vowels /ɪ/ and /æ/ (*impact*) and /ʌ/ and /ɛ/ (*upset*) in the sonority hierarchy. Sounds have different acoustic power – for instance, vowels are typically louder than consonants (O’Grady and Archibald, 2016). A low vowel like /æ/ is higher in the sonority hierarchy than a mid-high vowel like /ɪ/ and naturally louder, partly because it is pronounced with a larger mouth opening, as argued by Ladefoged and Johnson (2015) on the example of the low vowel /ɑ/ and the high vowel /i/. The increased intensity of the /ɪ/ vowel in the first syllable of *impact* is therefore perceived as relatively more salient and unusual compared to the pitch and duration conditions. While sonority has been defined as the loudness of a sound “relative to that of other sounds with the same length, stress, and pitch” (Ladefoged and Johnson, 2015, p. 255), more sonorous sounds are usually not only louder but also have higher pitch (O’Grady and Archibald, 2016) and intrinsically longer duration (Lehiste, 1970), so it is unclear whether the independent influence of intensity can be isolated. Conversely, it is also unclear to what extent listeners are influenced by the other common acoustic correlates and recover them even if only one cue is manipulated. In order to address these issues and improve the ecological validity of the stimuli, future studies can incorporate a relative manipulation of two cues in one stimulus. This can be achieved by manipulating two cues simultaneously, yet with a higher strength of one cue in relation to the other. This approach can yield additional insights after having shown that each of the cues is effective on its own.

One issue that requires attention is that further negativities following the MMN component were especially pronounced for the duration deviants (Figure 3). It seems likely that they are a result of an unavoidable physical confound: by necessity, the end of the word (i.e., the final /t/ sound) is shifted to a later timepoint for duration prolongation, whilst this is not the case for pitch and intensity deviants. The additional negativities might stem from temporal shifts of an ERP component elicited by the /t/ sound in the duration deviant, but not the standard nor the other deviants. Indeed, following the voiceless velar plosive /k/, the /t/ of /ɪmpækt/ can appear as a stop sound following a moment of silence, as visible in the spectrograms of the stimuli (Figure 1). The /t/ sound can thus evoke a distinct ERP component. Such physical stimulus differences are usually avoided in MMN research by control conditions in which the roles of standard and deviant are swapped (Jacobsen and Schröger, 2001, 2003), but this was unfeasible for the multi-deviant paradigm employed here (see Sandmann et al., 2010). Importantly, the physical difference pertains to both language groups alike and thus cannot induce or obscure any effects of interest; moreover, it happens well after the MMN latency range. Some longer-lasting (less transient) negative deflections were also observed in the other cue conditions; these could be late components like the LDN related to psycholinguistic processes in the perception of word stress changes, as in some of the previous studies described above (Honbolygó et al., 2017; Zeng et al., 2022). The LDN has been connected to more long-term representations such as a language-specific familiarity effect as well as to attention (Zeng et al., 2022).

Finally, while MMN studies are useful for exploring pre-attentive processing, their design requires the repetition of many standard and deviant trials per stimulus to establish a low probability of the deviant and elicit the MMN response (e.g., Fitzgerald and Todd, 2020). In order to incorporate six deviants (three stress cues and two stress positions), the current study featured 3,120 standards and 1,080 deviants and accordingly presented them as individual words without sentence context. However, word stress correlates are dependent on sentence stress, pitch accent and intonation (e.g., Dogil and Williams, 1999). Differences in word stress cue perception based on intonational context can be examined in follow-up behavioral studies. MMN studies help us identify what the (language) system of a listener is generally capable of, that is, whether the listeners can process the cue at all. Then, behavioral studies allow the integration of context and help us determine whether the listener can use the cue for language processing. Yet, as demonstrated in Domahs’ 2012 study on Polish word stress misplacement, there can be a dissociation between behavioral and EEG indicators of stress violation detection – although the success rate in the behavioral task was low, EEG provided evidence for successful word stress violation processing. Thus, the combination of behavioral methods with psychophysiological methods like EEG can generate valuable insights on perception and open up perspectives for applications in language teaching. The existence of an ERP effect indicates that the auditory system can process the cue and the training may need to focus on how to use it in perception and production – for instance, by paying attention to speaker variation. If there is no ERP effect, the training may need to focus more on acoustic aspects of the target cue and establish the non-native speech categories in the learner’s cognition before moving on to the cue’s use in perception and production.

### 4.2. Applications in language technologies and learning

It has been repeatedly demonstrated that the perception of non-native English speakers differs from that of native speakers (Chung and Bidelman, 2016; Zeng et al., 2022) and also between learner groups (Meng et al., 2020; the current study). Moreover, both phonetic and phonological factors affect word stress perception, as suggested in the current study. These differences speak against a one-size-fits-all approach in the design of English language products. Language technologies like text-to-speech systems need to be customizable to address the needs of different native and non-native speaker groups. Especially in educational scenarios with English as an instructional language, adapting a pedagogical agent to the learner groups has the potential of easing language processing and facilitating learning of subject matter in English. Such technologies could be informed by the users’ language systems and accordingly by which stress change will be salient to them. Prosody is crucial for synthesized speech since it contributes to the perception of artificial voices as more natural and likeable (Kühne et al., 2020; Ehret et al., 2021). Diversifying voice systems’ accentuation settings could thus have a positive effect on the processing of non-native artificial speech. Future studies can therefore explore strategies to best use insights on non-native word stress cues to customize artificial voice input that matches human sociolinguistic experiences. While we did not find any particular cues that were perceived more strongly by German and Slavic English learners, other groups like Mandarin and Cantonese speakers could benefit from stress cued through more pitch information, as inferred from previous studies (Tong et al., 2014; Meng et al., 2020; Zeng et al., 2022; Zhang et al., 2023). In addition, looking at applications for the teaching of English to speakers of other languages, all learners are likely to benefit from a language curriculum paying attention to both phonetic (syllable prominence) and phonological (syllable structure, lexical class, rhythm) factors in stress assignment. Language technologies like re-synthesis software can be integrated in the English classroom to demonstrate how the change of individual word stress cues like pitch, duration and intensity affects the perception of word stress. Still, the practical implementation of these ideas in the curriculum and supportive learning technologies is not straightforward and deserves additional research.

## 5. Conclusion

Research in word stress perception and production has indicated a partial insensitivity of Slavic language speakers to native and non-native word stress (e.g., Domahs et al., 2012). Based on EEG experiments with Slavic and German advanced learners of English, we found a robust MMN elicited at English lexical stress changes cued through pitch, intensity, and duration. The results indicate a pre-attentive detection of word stress changes in English as a non-native language cued through suprasegmental information. Comparisons between the language groups’ perception of the stress position found German speakers to have more differentiated response to second vs. first syllables than Slavic participants, ruling out stimulus effects. This tendency can be explained by the different rhythmic structure of the languages, where the syllable-timed characteristics of the Slavic languages drive their speakers to perceive the first syllable stressed ˈ*impact* as similarly salient to second syllable stressed *im*ˈ*pact*, even though the former carries the more frequent stress position in their native languages and in English. Intensity was more prominent in first syllable stressed deviants in comparison to pitch and duration, hinting at influences from the vowels’ place in the sonority hierarchy. The processes involved in non-native word stress perception have proven to be a complex combination of phonetic and phonological regularities which have opened promising opportunities for future research. The insights from this study can be applied in the customization of language technologies, which can incorporate the most salient cues for non-native speakers of English from different backgrounds and assist their perception. English language curricula should pay attention to both phonetic and phonological stress placement regulations to support non-native acquisition of word stress. Thus, English language products can adapt to real-world perceptual variation in cue weighting.

## Data availability statement

The pre-processed EEG data (single-subject averages) supporting the conclusions of this article are available alongside with the analysis code in a publicly accessible repository: https://osf.io/sb6pv/. The raw EEG data will be made available by the authors upon request, without undue reservation.

## Ethics statement

All study procedures were reviewed and approved by the Ethics Committee of Chemnitz University of Technology (case no. 101560561). The participants provided their written informed consent to participate in this study.

## Author contributions

MI, JS, and AB contributed to the conception and design of the study. MI and AB prepared the experimental stimuli and programmed the code for stimulus presentation. MI and CN collected and analysed the data. AB reviewed the data analysis. MI wrote the first draft of the manuscript. JS and AB were responsible for funding acquisition and supervision. All authors contributed to the manuscript revision, read, and approved the submitted version.

## Funding

This research was funded by the Deutsche Forschungsgemeinschaft (DFG, German Research Foundation) – Project-ID 416228727 – SFB 1410 TP A04 (AB) and TP D03 (JS). This work was also supported by a PhD scholarship awarded to MI by the State of Saxony.

## Conflict of interest

The authors declare that the research was conducted in the absence of any commercial or financial relationships that could be construed as a potential conflict of interest.

## Publisher’s note

All claims expressed in this article are solely those of the authors and do not necessarily represent those of their affiliated organizations, or those of the publisher, the editors and the reviewers. Any product that may be evaluated in this article, or claim that may be made by its manufacturer, is not guaranteed or endorsed by the publisher.

## Footnotes

^1^Here we only look at languages represented in our participant sample for the sake of brevity.

^2^Surprisingly, in contrast to the Cambridge English Dictionary (2023) which only lists /Imˈpækt/ as pronunciation transcription of the verb *impact*, the Oxford English Dictionary (2023) lists both /Imˈpæk(t)/ and /ˈImˈpæk(t)/ as possible pronunciations of the verb. The alternative pronunciation is most likely due to possible rhythm-based stress shifts.

^3^One participant was omitted due to a lacking response to the question on *years studied English*, therefore *df* = 29.
